# Anti-Phytopathogenic Activities of Macro-Algae Extracts

**DOI:** 10.3390/md9050739

**Published:** 2011-05-03

**Authors:** Edra Jiménez, Fernando Dorta, Cristian Medina, Alberto Ramírez, Ingrid Ramírez, Hugo Peña-Cortés

**Affiliations:** 1 Biotechnology Center “D. Alkalay L.”, Universidad Técnica Federico Santa María, Avda. España 1680, Valparaiso, Chile; E-Mails: edra.jimenez@usm.cl (E.J.); fernando.dorta@usm.cl (F.D.); ingrid.ramirez@usm.cl (I.R.); 2 Fundación Chile, Avda. Parque Antonio Rabat Sur 6165, Vitacura, Santiago, Chile; E-Mails: cmedina@fundacionchile.cl (C.M.); aramirez@fundacionchile.cl (A.R.)

**Keywords:** macro-algae, plant pathogen, *Botrytis cinerea*, *Phytophthora cinnamomi*, tobacco mosaic virus (TMV)

## Abstract

Aqueous and ethanolic extracts obtained from nine Chilean marine macro-algae collected at different seasons were examined *in vitro* and *in vivo* for properties that reduce the growth of plant pathogens or decrease the injury severity of plant foliar tissues following pathogen infection. Particular crude aqueous or organic extracts showed effects on the growth of pathogenic bacteria whereas others displayed important effects against pathogenic fungi or viruses, either by inhibiting fungal mycelia growth or by reducing the disease symptoms in leaves caused by pathogen challenge. Organic extracts obtained from the brown-alga *Lessonia trabeculata* inhibited bacterial growth and reduced both the number and size of the necrotic lesion in tomato leaves following infection with *Botrytis cinerea.* Aqueous and ethanolic extracts from the red-alga *Gracillaria chilensis* prevent the growth of *Phytophthora cinnamomi*, showing a response which depends on doses and collecting-time. Similarly, aqueous and ethanolic extracts from the brown-alga *Durvillaea antarctica* were able to diminish the damage caused by tobacco mosaic virus (TMV) in tobacco leaves, and the aqueous procedure is, in addition, more effective and seasonally independent. These results suggest that macro-algae contain compounds with different chemical properties which could be considered for controlling specific plant pathogens.

## 1. Introduction

Like animals, plants are also exposed to a wide variety of enemy organisms, which can damage their tissues. These organisms include insect pests, nematodes, pathogenic fungi, bacteria, viruses, and many other organisms, which can cause a severe reduction in the quantity as well as the quality of the crops. Although several strategies have been developed in recent years for controlling plant pathogens, many plant pathogens and the insects that often spread them have overcome some pesticides, agricultural practices and biocontrol that previously held them in check [1,2]. Efficient global travelling is spreading viral, bacterial, and fungal pathogens into new areas, while global warming is allowing insect vectors to expand their ranges. Until now, breeding for pathogen resistance and spraying fungicides have been the main recommended measures for controlling the disease, but the current interest in the environment and human health has intensified the development of new alternative control methods. Therefore, alternative disease management using natural compounds and other resistance types needs to be considered to inhibit the growth of plant pathogens or to act on plant tissues as elicitor of the plant defense mechanism in order to exploit renewable resource(s) for crop protection in agriculture.

Marine algae represent a great source of a vast variety of complex natural products and could be a promising source of a novel bioactive compound that can help plant survival by offering protection against stress imposed by pathogens. Marine algae may have several applications in agriculture [3,4]. It has been reported that seaweeds possess compounds exhibiting antimicrobial potential against the pathogenic microbes of medical, agricultural, and environmental importance. Thus, antiviral, anthelmintic, antifungal, and antibacterial activities have been detected in green, brown, and red algae [5–8]. There are numerous reports on the biological activities of macroalgae against human pathogens, fungi, and yeast [6]. However, studies related to phytopathogens are being restricted to pathogens of commercial crops such as tobacco, rice, and citrus trees [9–12]. Some substances extracted from seaweeds have antibacterial actions and other properties including antifungal activities and growth inhibition of plants [13–18].

Plants cannot move to run away from environmental challenges. Biotic stresses result from a series of potential pathogens such as fungi, bacteria, nematodes, insects, and viruses, causing significant yield losses in crops. Some of the fungal, bacterial, and viral plant pathogens are highly specific, infecting only a single crop or host species. Other pathogens are more opportunistic, infecting a broad range of susceptible hosts [19–21]. Gram negative bacteria such as *Pseudomonas syringae* and *Erwinia carotovora* use a large and well-defined repertoire of effector proteins to cause disease in tomato, Arabidopsis and potato plants among others [22–24]. Fungal plant pathogens such as *Botrytis cinerea* and *Phytophthora cinnamomi* attack a wide range of agriculturally and ornamentally important plants [25,26].

In this report, we evaluate the effects of aqueous and ethanolic crude extracts from nine different seaweeds collected on the coastline of Chile in different seasons on plants pathogens such as the bacteria *Pseudomonas syringae*, *Erwinia carotovora*, the fungal pathogens *Phythophthora cinnamomi* and *Botrytis cinerea*, and on the tobacco mosaic virus (TMV).

## 2. Results and Discussion

Aqueous and 50% ethanolic extracts were obtained from nine different Chilean seaweeds collected on the coastline during the years 2008–2009 (Table 1). The algae were collected in four different year periods to verify the seasonal dependence of yield and of the putative active compound isolated under the experimental conditions.

### 2.1. Antimicrobial Activity

**Figure 1.**
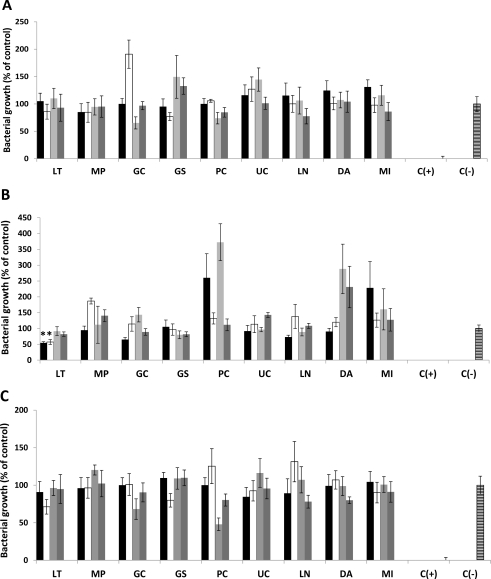

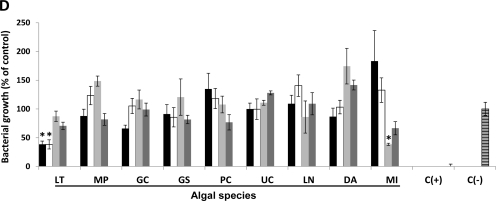
Effect of extracts obtained from Chilean seaweeds on growth of *Erwinia carotovora* and *Pseudomonas syringae*. Crude aqueous (**A** and **C**) and 50% ethanolic extracts (**B** and **D**) isolated from *Lessonia trabeculata* (LT), *Macrocystis pyrifera* (MP), *Gracilaria chilensis* (GC), *Gigartina skottsbergii* (GS), *Porphyra columbina* (PC), *Ulva costata* (UC), *Lessonia nigrescens* (LN), *Durvillaea antarctica* (DA) and *Macrocystis integrifolia* (MI) collected in season 1 (black boxes), season 2 (white boxes), season 3 (light gray boxes) and season 4 (dark gray boxes) at a concentration of 10,000 ppm were incubated with *E. carotovora* (**A** and **B**, respectively) or with *P. syringae* (**C** and **D**) as described in Experimental Section. Negative control [C(–)] represents bacteria growing in media without algae extracts whereas positive control [C(+)] corresponds to bacteria growing in media containing 5 μM streptomycin. The activities of the extracts were evaluated in microculture assays by growing bacteria as described in the Experimental Section. All values represent mean of triplicate determinations ± standard deviation. Significant differences (*P* < 0.05) from control cell cultures are marked with an asterisk.

The algal extracts were tested for inhibitory activity against two plant pathogenic strains: *Erwinia carotovora* and *Pseudomonas syringae*. Extracts in different concentrations were incubated with the bacteria in the corresponding growth media. Aqueous extracts obtained from all algae, independently of the season, did not alter bacterial growth (Figure 1A,C). From the ethanolic extracts, only those obtained from *L. trabeculata* collected in summer (season 1) and autumn (season 2) showed an inhibitory effect of around 40–60% in comparison to the control ones—against both *E. carotovora* (Figure 1B) and *P. syringae* (Figure 1D)—whereas those obtained from *M. integrifolia* in springtime (season 3) partially reduced (around 50% of the negative control) the growth of *P. syringae* (Figure 1D). Such effects were observed solely by using a medium containing high extract concentration (10,000 ppm). Lower amounts of extracts in the growth medium did not show an inhibitory effect on bacterial growth. Similarly, the presence of higher extract concentration in the medium did not increase the observed inhibitory effect (data not shown). The results suggest that active compounds are present in LT and MP ethanolic extracts. The extracts are able to affect bacterial growth and their activity is dose- and season- dependent.

### 2.2. *In Vitro* Antifungal Activity

**Figure 2.**
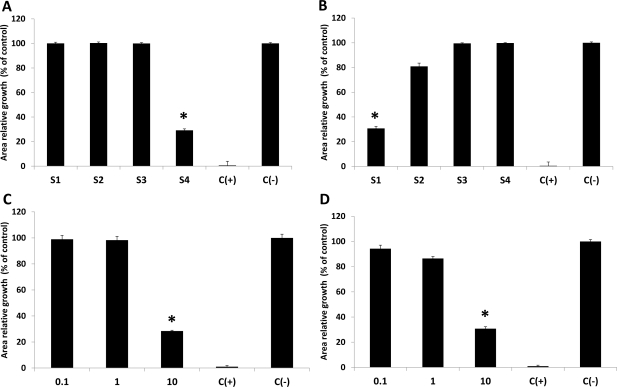
Effect of algae extracts on growth of *Phytophthora cinnamomi*. Fungal mycelium was grown on medium containing 10,000 ppm either of (**A**) aqueous or (**B**) ethanolic extracts obtained from red alga *Gracillaria chilensis* in four different seasons (S1: summer; S2: autumn; S3:spring; S4: spring–summer) as described in Experimental Section. Different concentrations (0.1 = 100 ppm; 1 = 1000 ppm and 10 = 10,000 ppm) of the (**C**) active aqueous extracts obtained from samples collected in season S4. Ethanolic extracts obtained in S1 were also tested (**D**). Negative control [C(–)] represents mycelium growing in media without algae extracts whereas positive control [C(+)] corresponds to fungal growing in media containing 400 ppm Metalaxil. Activities of the extracts were evaluated as described in Experimental Section. All values represent the mean of triplicate determinations ± standard deviation. Significant differences (*P* < 0.05) from control fungal cultures are marked with an asterisk.

Examining the effect of the obtained aqueous and ethanolic extracts from all collected algae on the growth of *P. cinnamomi* or *B. cinerea* by using the agar-diffusion assay technique demonstrated that most of them do not alter the development of either fungi (data not shown), suggesting either that they are not able to diffuse across the agar or that the extracting condition does not allow the isolation of compounds with properties to alter fungal growth under these conditions. Aqueous or ethanolic extracts obtained solely from *G. chilensis* led to a reduction of the growing capacity of *P. cinnamomi* in a dose- and season-dependent manner (Figure 2). Aqueous extracts obtained from samples collected, particularly in the spring–summer samples, (season 4) showed an effect on *P. cinnamomi* growth (Figure 2A) whereas the ethanolic extracts obtained from samples collected in summer (season 1) seem to have a similar effect on the growth of *P. cinnamomi* mycelium (Figure 2B). Both are active in concentrations of 10,000 ppm, reducing *P. cinnamomi* development in around 50% compared to the negative control (Figure 2A,C). Both extracts did not show such inhibitory effects for decreased concentrations (Figure 2C,D). The results suggest the presence of bioactive compounds which are able to block the growth of *P. cinnamomi* in both types of extracts. It remains unknown whether similar compounds are present in the season 4 aqueous separates or in the season 1 ethanolic isolated extracts. However, the active elements seem to be produced or accumulated in the algae in the spring–summer period and may have water soluble properties because ethanolic extracts obtained at a higher alcoholic grade were not able to inhibit *P. cinnamomi* mycelium growth (data not shown).

### 2.3. *In Vivo* Antifungal Activity

**Figure 3.**
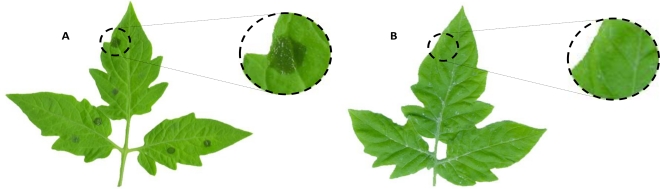
Effect of algae extracts on tomato leaves following infection with *Botrytis cinerea*. Tomato leaves were used for evaluating the protecting properties of Chilean algae extracts. Control leaves without previous treatment were infected with fungal conidial suspension. Another set of tomato leaves were treated with different concentrations of either aqueous or ethanolic extracts before pathogen challenge as described in Experimental Section. The picture represents an average example of (**A**) control leaves and (**B**) leaves treated with commercial fungicide Captan before to *B. cinerea* infection.

**Figure 4.**
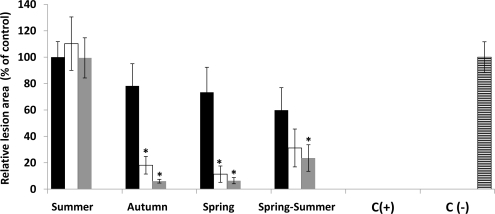
Protecting effect of ethanolic extracts from *Lessonia trabeculata* on tomato leaves challenged with *Botrytis cinerea*. Tomato leaves were treated before pathogen challenge with solutions containing 1000 (black boxes), 5000 (white boxes) or 10,000 (light gray boxes) ppm of ethanolic extracts obtained from different seasons (summer, autumn, spring, and spring–summer). Negative control [C(–)] represents *B. cinerea* mycelium growing on leaves without previous treatment with extracts whereas positive control [C(+)] corresponds to fungal growing on leaves which were pre-treated with solutions containing 600 ppm of Captan. Activities of the extracts were evaluated as described in Experimental Section. All values represent the mean of triplicate determinations ± standard deviation. Significant differences (*P* < 0.05) from negative control leaves are marked with an asterisk.

In order to examine whether the extracts have some properties to protect plant leaves against infection with *B. cinerea*, tomato petioles were solvent treated (Figure 3A) or pre-treated with either aqueous or ethanolic extracts at different concentrations before pathogen challenge (Figure 3B). None of the aqueous extracts from the collected algae caused any reduction in injury severity in leaves after pathogen infection (data not shown). Similar results were obtained with ethanolic extracts (data not shown). However, ethanolic extracts from *L. trabeculata* collected in three different seasons reduced the damage in tomato leaves caused by *B. cine*rea infection (Figure 4). Extracts gained from samples collected in seasons 2, 3 and 4 seem to be more efficient in providing the protective effect, resulting in a reduction of both the number and the size of lesions caused following the infection with the pathogen compared to negative control leaves. The protective capacity of these extracts is more effective at 10,000 ppm, reaching a protection grade of 95% in leaves treated with extracts of season 2, 93% with extracts of season 3 and 72% in those treated with extracts of season 4 (Figure 4B). Lower concentrations of the extracts isolated from season 2 and 3 also reduced the damage in tomato leaves, in around 80% compared to the injury observed in negative control leaves (Figure 3B). The results suggest that LT ethanolic extracts contain certain active principle(s) which may provide protection to tomato leaves against the pathogenic fungi *B. cinerea* in a dose- and seasonal dependent manner.

### 2.4. *In Vivo* Antiviral Activity

**Figure 5.**
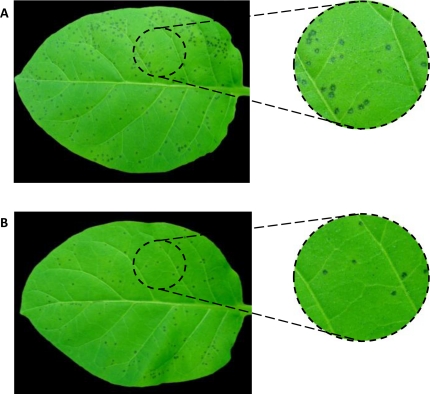
Effect of algae extracts on the infection symptoms in tobacco leaves caused by tobacco mosaic virus (TMV). *In vivo* assays using tobacco leaves were performed to determine the influence of macroalgae extracts on tobacco leaves challenged with TMV. Therefore, (**A**) non-treated tobacco leaves or (**B**) treated ones with solutions containing different amounts of *D. antarctica* extracts were subsequently infected with TMV. Damage symptoms were evaluated as described in Experimental Section.

**Figure 6.**
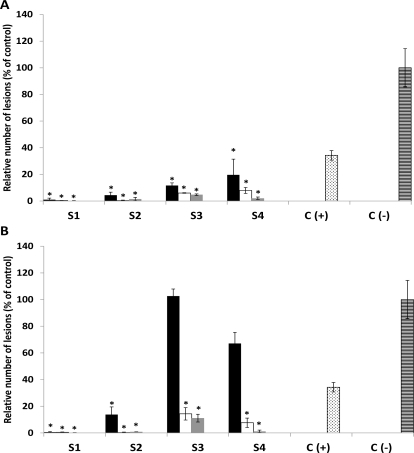
Protective effect of *D. antarctica* extract on tobacco leaves following TMV infection. Leaves of tobacco plants were treated with algae extracts from samples collected at different times (S1–S4), with Ribavirin [C(+), dotted boxes] or non-treated [C(–), narrow horizontal boxes] before infection with TMV. (**A**) Aqueous and (**B**) ethanolic extracts were applied by spraying at different concentrations (black boxes: 1000 ppm; white boxes: 5000 ppm; gray boxes: 10,000 ppm) and the activities of the extracts were evaluated as described in Experimental Section. All values represent the mean of triplicate determinations ± standard deviation. Significant differences (*P* < 0.05) from negative control leaves are marked with an asterisk.

**Figure 7.**
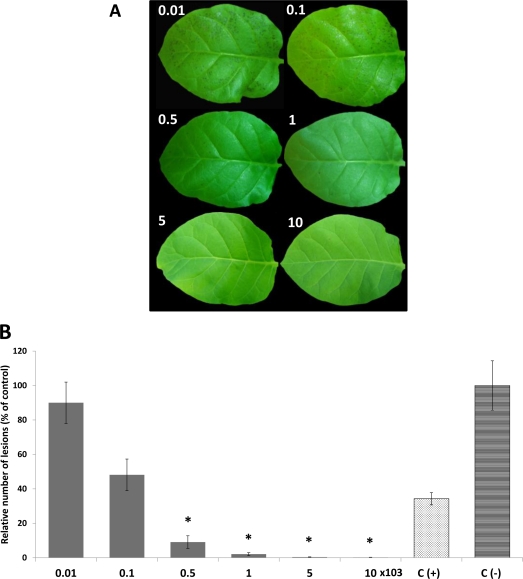
Protective effect of algal aqueous extracts on tobacco. Leaves of tobacco plants were treated with different concentrations ((0.01–10) × 10^3^) of aqueous extracts obtained from samples collected in season 1 before infection with TMV (A). Negative control [C(–), narrow horizontal box] represents non-treated tobacco leaves whereas positive control [C(+), dotted box] corresponds to those pre-treated with Ribavirin. Activities of the extracts were evaluated as described in *Experimental Section*. All values represent the mean of triplicate determinations ± standard deviation. Significant differences (*P* < 0.05) from control leaves are marked with an asterisk.

Most of the extracts obtained from Chilean algae under the experimental conditions did not show a protecting effect on tobacco leaves from damage caused following TMV infection (data not shown). Nevertheless, both extracting conditions provided crude extracts from the alga *Durvillea antarctica* which reduced the damage symptoms in tobacco leaves produced following TMV challenge. The protecting effect presented by both extracts led to a reduction of the number and the size of necrotic lesions (Figure 5A,B). This effect resulted in all aqueous or ethanolic extracts when 5000 and 10,000 ppm were applied independently of collecting time. The reduction of the injury severity caused following TMV infection was higher than 90% compared to those detected in the negative controls (Figure 6A,B). The protective effect provided by the extracts is superior to those obtained by applying the commercial antiviral Ribavirin. The protective effect decreased with lower concentrations, the lowest one being found in ethanolic extracts from seasons 3 and 4 (Figure 6B). The most important protective effect was observed by applying extracts from season 1 or season 2 independently of the extracting procedure. Both extracts are active even at amounts of 1000 ppm and the disease symptoms are more severely reduced by applying extracts obtained from algae collected in season 1 (Figure 6A,B). Application of 100 ppm already reduces necrotic lesions (Figure 7A) in infected leaves, reaching a protective result of 95% compared to the negative control at concentrations of 500 ppm (Figure 7B).

## 3. Experimental Section

### 3.1. Algal Materials

*Durvillaea antarctica* (DA), *Gracilaria chilensis* (GC), *Gigartina skottsbergii* (GS), *Lessonia nigrescens* (LN), *Lessonia trabeculata* (LT), *Macrocystis integrifolia* (MI), *Macrocystis pyrifera* (MP), *Porphyra columbina* (PC), and *Ulva costata* (UC) were collected from the Chilean coast during four different periods distributed in summer (season 1, December 2008–January 2009), autumn (season 2, April–May 2009), spring (season 3, October 2009) and spring–summer (Season 4, November–December 2009). After collecting, all samples were washed thoroughly under tap water, packed in plastic bags, transported under cool conditions with dried ice to the laboratory and stored at −80 °C until extracts were prepared.

### 3.2. Preparation of Aqueous and Ethanolic Extracts

Aqueous extractions were carried out as described in the literature [4,27] with some modifications. Seaweed samples (60 g) were ground into powder under liquid nitrogen using a pestle and mortar. Aqueous extracts were prepared by increasing the temperature of the 50 mL of water to 85–90 °C and maintaining it for 1 h with constant magnetic stirring. The mixture was filtered and finally centrifuged at 6000 rpm for 20 min at room temperature. Supernatant were pooled together and evaporated under reduced pressure using a rotary evaporator at 50 °C until 1/4 of the volume. Concentrated aqueous extracts were freeze-dried and stored at 4 °C until biological assays. 50% aqueous ethanol extractions were carried out as described in the literature [28–30] with some modifications. The algae powder (60 g) was extracted with 200 mL of 50% aqueous ethanol under magnetic stirring for 24 h at room temperature in dark conditions. A fraction of this extract remained insoluble and was removed by filtration. The ethanolic extract was stored in bottles light protected. A second extraction was prepared with other 200 mL of 50% aqueous ethanol from the same insoluble material. Two extracts of each sample were pooled together and evaporated under reduced pressure using rotary evaporator at 40 °C. The 50% aqueous ethanol extracts were finally dried in a desiccator under a vacuum using blue silica gel as a desiccant. Dried extracts were stored at 4 °C until biological assays.

### 3.3. Plant Material

Tomato plants (*Solanum esculentum*, cv. Patron) were obtained from seed germination and grown for eight weeks under a 10 h at 25 °C/14 h at 18 °C light/dark cycle and 70% relative humidity in a greenhouse. Seeds of tobacco, *Nicotiana tabacum* L. (cv. Xanthi-nc, NN genotype); were sown in soil: vermiculite mixture (3:1) and grown at 22–24 °C in growth chambers programmed for 16-h light (cool-white fluorescent lamp/200 μmol·m^−2^·s^−1^) and 8-h dark cycle.

### 3.4. *In Vitro* Antibacterial Activity Assays

Liquid-dilution methods were used to evaluate the effect of ethanolic and aqueous extracts on the growth of *Erwinia carotovora* (NCPPB 312) and *Pseudomona syringae* (NCPPB 281). Bacteria were grown in sterile tubes with 6 mL of Müeller-Hinton medium and incubated at 27 °C for 12 h with shaking to produce an initial culture. The antimicrobial activity of Chilean seaweed extracts was evaluated by observing the growth response of both micro-organisms in samples with different concentrations of either aqueous or ethanolic extracts [31–33]. All assays were realized on sterile 96-well microplates with a final volume of 200 μL containing Müeller-Hinton medium inoculated with aliquots of 1 μL of bacterial suspension (10^5^–10^6^ UFC/mL, initial culture) in the presence of different concentrations of algal extracts (10, 100, 1000, 5000 and 10,000 ppm). A Müeller-Hinton medium was utilized as a negative control [C(–)] and a Müeller-Hinton medium with 5 μM Streptomycin [34] was utilized as positive control [C(+)]. They were incubated for 6 h at 27 °C. Bacterial growth was monitored by measuring the optical density at 595 nm with a microplates reader every hour. All tests were performed in triplicate for each microorganism evaluated. Bacterial growth was shown as the arithmetic average expressed in terms of negative control (100%).

### 3.5. *In Vitro* Fungicide Activity Assays

A virulent isolate of *Botrytis cinerea* obtained from naturally infected grape berries [35] was prepared and kept by plating on potato dextrose agar at 5 °C. *Phytophthora cinnamomi* was gently provided by Dr. P. Sepúlveda from National Institute of Agricultural Research (INIA, La Platina, Santiago, Chile). Agar-diffusion technique was used to evaluate the effect of aqueous and ethanolic extracts on the growth of *Phytophthora cinnamomi* [36,37] and *Botrytis cinerea* [38,39]. Fungicide activity assays of the extracts were evaluated in microcultures by growing the fungus in sterile 12-well microplates at a final volume of 2 mL medium containing different extract concentrations. Clarified V8 (Campbell Soup) medium containing Metalaxil [40] for *P. cinnamomi* or PDA medium for *B. cinerea* with commercial fungicide Captan [41,42] was utilized as positive control [C(+)] or, without fungicide, as negative control [C(–)]). The medium in each slot was then inoculated with a small block (4 mm) of clarified V8 or PDA medium containing fungal hyphae excised from the edge of an actively growing culture. Mycelium growth was evaluated visually and measured after 48 h of incubation at room temperature. Each treatment was independently performed in triplicate.

### 3.6. Virus Bioassay

The antiviral activity was determined by infecting tobacco plants with tobacco mosaic virus (TMV) according to Enyedi *et al.* [43]. Leaves of 6- to 8-week-old plants, sprayed with different concentrations of seaweed extracts, Ribavirin [44–46] or solvent control, were abraded with wet carborundum (400 grit) and inoculated with a 200 μL suspension of TMV (Deutsche Sammlung von Mikroorganismen und Zellkulturen GmbH (DSMZ), PV-0175) (25 μg/mL in 50 mM phosphate buffer pH 7.5) by gently rubbing the adaxial leaf surface. The leaves were rinsed with deionized water following inoculation. The mock-leaves were abraded and inoculated only with a phosphate buffer. After inoculation, the plants were maintained at 22–24 °C under growth chamber conditions. Leaf lesions were measured and imported into R environment software [47] in order to carry out statistical analysis.

### 3.7. *In Vivo* Assays in Tomato Leaves with Botrytis Cinerea

For the preparation of a *B. cinerea* inoculum, fungus was grown at 20 °C under a diurnal light regime (12/8 light/darkness photoperiod) photoperiod for 6–7 days. For harvesting, plates were superficially washed twice with sterile water to extract conidia using a glass rod. Then aqueous spore collections were put into a blender with a few drops of Tween 20. Spore suspension was adjusted to one million per ml and then transferred into a spraying device producing a very thin droplet. Tomato plant leaves (petioles) were utilized to test the antifungal activity of ethanol and aqueous seaweed extracts. Detached tomato leaves were transferred to closed plastic boxes containing wet absorbent paper to provide a 95% to 100% humidity, theessential condition to achieve spreading lesions [48]. Four milliliters of extract at different concentrations were sprayed on each tomato petiole. The control set was treated either with water alone (negative control) or water containing the commercial pesticide Captan (positive control). Two hours after treatment the tomato petioles were infected with *Botrytis cinerea* by depositing 10 μL of the pathogen conidial suspension (3.5 × 10^7^ spores/mL) on the leaf surface. Two days after inoculation, necrotic spots on the leaves were calculated by scanning, using an Epson Perfection Photo 3940 scanner (Epson, Long Beach, CA, USA). The generated images were recorded as black-and-white .tiff files, and processed with the Area Density tool from GelPro Analyzer software (Media Cybernetics Inc., Sarasota, FL, USA). The dark areas were translated into pixels. Data were then exported into MS-Excel (Microsoft) files and then processed for analyses of variance and means. This analysis was performed separately applying an LSD test at the 5% level of significance using Statgraphics Plus 5.1 (Manugistics Inc., Rockville, MD, USA).

### 3.8. Statistics

The following approach was used to determine significant differences between the different treatments and their control. A one-way ANOVA was performed to identify significant differences among the treatments-control groups. For a significant statistical test (*P* < 0.05), Tukey’s honesty significance test was applied to compare the means of every treatment against the control and simultaneously establish their significance (*P* < 0.05). All data were presented as mean ± standard deviation (mean ± S.D.). Significant *P*-values were indicated with stars (* *P* < 0.05, ** *P* < 0.01, *** *P* < 0.001). All experiments were performed independently three times.

## 4. Conclusions

Several reports have demonstrated that organic crude extracts from red algae show antiprotozoal and antimycobacterial activities [49], whereas other fractions containing sesquiterpenes are active against fish and human pathogenic bacteria [5,50–52]. Green algae also have certain sesquiterpenes compounds which are active against human and marine aquacultural pathogens [5,50,53–55]. Substances isolated from brown algae also show properties to inhibit the growth of marine bacteria, fungi, and mussels [56]. Marine macroalgae have also been reported to have specific small molecules with antimicrobial chemical defenses against algal pathogens [57–59].

Other studies have also demonstrated that separates obtained from some macroalgae by organic solvent allow the control of certain plant pathogenic bacteria [15,60]. Similar to these studies, our results demonstrate that ethanolic extracts provide a system of isolating biologically active compounds from *L. trabeculata* with biological properties to reduce the growth of both studied gram negative plant pathogenic bacteria.

Antifungal compounds have been detected in different algal species. For example, extracts of the brown alga *Cystoseira tamariscifolia* showed *in vitro* fungal activity against the plant pathogens *B. cinerea, F oxysporum* and *Verticillium album-atrum* [61] as well as the food spoilage *Aspergillus* spp. [17,62]. In our study, aqueous and ethanolic extracts from *G. chilensis* contain active compounds which are able to reduce the growth of *P. cinnamomi* under *in vitro* conditions whereas ethanolic separates from *L. trabeculata* reduced the disease severity caused by *B. cinerea* when sprayed on tomato leaves. Thus, ethanol-soluble extracts from two different algae contain active compound(s) having polar characteristics which can act directly on the mycelial growth of *P. cinnamomi* or activating plant defense mechanisms to protect the tissue against *B. cinerea* infection. Preliminary efforts to characterize the active components of the extracts, including column chromatography and biological activity approaches, demonstrate that the active fraction contains substances different to polysaccharides or proteic nature (data not shown).

Antiviral activities have also been described for compounds related to sulfated polysaccharides [7] obtained from red [17,63,64] and green algae [65–67]. In our study, all the aqueous and ethanolic extracts obtained from the alga *Durvillaea antarctica*, independently of collecting time, protectedthe tobacco leaves against infection with TMV when applied at high concentrations. When lower amounts were applied to the leaves, aqueous and ethanolic extracts from samples collected in spring and summer were more efficient, suggesting that the main effective components in these extracts were polysaccharides and that their production in algal tissue depends on collecting time.

Inhibitory activity of extracts against plant pathogens were commonly found in either aqueous or ethanolic extracts obtained from algae collected in the summer or spring–summer season. In the case of the extracts obtained from *G. chilensis* and *D. antarctica* a co-relation between the season dependence and a higher anti-pathogenic activity of the extracts can be observed. The most effective control on *P. cinnamomi* or *B. cinerea* growth resulted by using high extract concentrations (10,000 ppm), suggesting a low biological activity of the extracts which might be given due to a low amount of the active components in the sample, due to the presence of some inhibitory substances which reduce the antifungal activity or a limited capacity of the extraction procedure for the isolation of the components with antifungal activities. In contrast to this, antiviral effect on tobacco leaves can be observed by the application of aqueous or ethanolic extracts of *D. antarctica* extracts at much lower concentrations, indicating a major effectiveness of the extraction process in gaining the active components against TMV.

Additional studies need to be performed to define and characterize, at the chemical and biochemical level, the preferential effect of algae extracts on microorganisms that damage many plants. Finally we conclude that the Chilean coast is a source of bioactive compounds with potential applications in agriculture, revealing activity to control plant pathogens.

## Figures and Tables

**Table 1.** Macroalgae collected at Chilean cost in four different periods during year 2008–2009.

**Name**	**Abbreviation**	**Type**	**Location**	**Season 1** **Summer**	**Season 2** **Autumn**	**Season 3** **Spring**	**Season 4** **Spring–Summer**
*Macrocystis pyrifera*	MP	Brown	Punta Arenas	Jan 2009	Apr 2009	Sept 2009	Nov 2009
*Macrocystis integrifolia*	MI	Brown	Puerto Aldea	Jan 2009	May 2009	Sept 2009	Nov 2009
*Lessonia nigrescens*	LN	Brown	Puerto Aldea	Jan 2009	May 2009	Sept 2009	Dec 2009
*Lessonia trabeculata*	LT	Brown	Puerto Aldea	Jan 2009	May 2009	Sept 2009	Nov 2009
*Durvillaea antarctica*	DA	Brown	Matanzas	Feb 2009	May 2009	Sept 2009	Dec 2009
*Gracilaria chilensis*	GC	Red	Quetalmapu	Dec 2008	Apr 2009	Sept 2009	Dec 2009
*Porphyra columbina*	PC	Red	Punta Arenas	Jan 2009	Apr 2009	Sept 2009	Dec 2009
*Gigartina skottsbergii*	GS	Red	Punta Arenas	Jan 2009	Apr 2009	Sept 2009	Dec 2009
*Ulva costata*	UC	Green	Coliumo	Jan 2009	Apr 2009	Sept 2009	Nov 2009
